# Genetic diversity and population structure of *Meretrix petechialis* in China revealed by sequence-related amplified polymorphism markers

**DOI:** 10.7717/peerj.8723

**Published:** 2020-03-25

**Authors:** Qiaoyue Xu, Junhong Zheng, Hongtao Nie, Qingzhi Wang, Xiwu Yan

**Affiliations:** 1 College of Fisheries and Life Science, Engineering Research Center of Shellfish Culture and Breeding in Liaoning Province, Dalian Ocean University, Dalian, China; 2 Liaoning Ocean and Fisheries Science Institute, Dalian, Liaoning, China

**Keywords:** *Meretrix petechial*, Genetic diversity, Polymorphism, SRAP, Genetic structure

## Abstract

Genetic variation in nine stocks of *Meretrix petechialis* collected from China was analyzed using sequence-related amplified polymorphism (SRAP) markers. Eight primer pairs produced 132 polymorphic loci with an average of 16.5 loci per primer pair. A population from Jiangsu had the highest percentage of polymorphic loci at 27.27%, suggesting that these resources had a rich genetic diversity. The Nei’s gene diversity of the nine populations ranged from 0.0647 to 0.0793; a population from Shandong was the lowest and a population from North Korea the highest. The Shannon’s information index was between 0.1023 and 0.1202, with the lowest in the Shandong population and the highest in the Jiangsu population. The Nei’s unbiased genetic distance between the nine populations was 0.0243–0.0570 and the genetic similarity was 0.9446–0.9760; the genetic distance between Guangxi and Shandong populations was the furthest (0.0570) and the genetic distance between Shandong and Jiangsu populations was the closest (0.0243). Nei’s gene diversity analysis indicated that the genetic variance was mainly found within individual geographical populations, and the analysis of molecular variance revealed low but significant genetic differentiation among local and regional populations. The limited gene flow (Nm = 0.555) was inferred as a major reason for the extent of genetic differentiation in *M. petechialis*. The results obtained here indicated that *M. petechialis* have high degree of genetic diversity and the potential of further breeding with excellent germplasm resources. This study provides a scientific basis for the protection of germplasm resources and the breeding of *M. petechialis*.

## Introduction

*Meretrix petechialis* is a marine shellfish species of economic and medicinal value that is mainly distributed and cultured in coastal China. In recent years, the scale of breeding in *M. petechialis* has resulted in an increasing demand for seedlings and the artificial seeds transplantation (Shen et al., 2003; Chen et al., 2004), which has affected the protection and development of *M. petechialis* germplasm resources. Therefore, the genetic diversity of *M. petechialis* germplasm resources can be determined to compare the genetic differences of different geographical populations, which can provide useful information for the protection of *M. petechialis* germplasm resources and breeding.

SRAP is a PCR-based molecular marker proposed by Li & Quiros (2001), which can be used for differential analysis of genomic DNA and cDNA (Li & Quiros, 2001). SRAP loci are derived from a single forward primer and numerous reverse primers, providing an efficient protocol for the discovery of polymorphisms. Upstream primers can specifically bind to the exon or promoter regions of genes, downstream primers can be specific to intron pairing. A SRAP marker is used to amplify an ORF in the genome and produces polymorphic amplification products due to the different lengths of introns, promoters and spacers in different individuals (Zhang et al., 2010).

Over the past few decades, the SRAP marker has been widely used in plant research, such as genetic map construction, genetic diversity analysis and comparative genomics (Tang et al., 2003). Meanwhile, SRAP was widely used to study germplasm resources and genetic diversity of species because of their special primer design in addition to their simplicity, stability and repeatability. In aquatic animals, Ding, Cao & Cao (2008) obtained *Ctenopharyngodon idellus* germplasm molecular markers by SRAP markers, as similarly reported in *Macrobrachium nipponense* (Qiao et al., 2012). In bivalves, the SRAP technique has been used to analyze differential gene expression in mantle with different external shell color in *Hyriopsis cumingii* (Wen et al., 2014) and *M. petechialis* (Zheng, Nie & Yan, 2019). In addition, Jin et al. (2013) established a SRAP–PCR system and analyzed the genetic diversity in Manila clam *Ruditapes philippinarum* with white, black and orange shell colors. However, there are very few studies on the genetic diversity of *M. petechialis* using sequence-related amplified polymorphism (SRAP) markers (Zhang et al., 2010). In this study, SRAP markers was used to analyze the genetic diversity and population structure of nine geographic populations of *M. petechialis* to reveal their levels of genetic differentiation, which is of great significance for the conservation and scientific management of genetic resources. Meanwhile, this work provided data about genetic polymorphisms and potential genetic improvements, which are critical information for selective breeding.

## Materials and Methods

### Collection of samples and extraction of DNA

**Figure 1:**
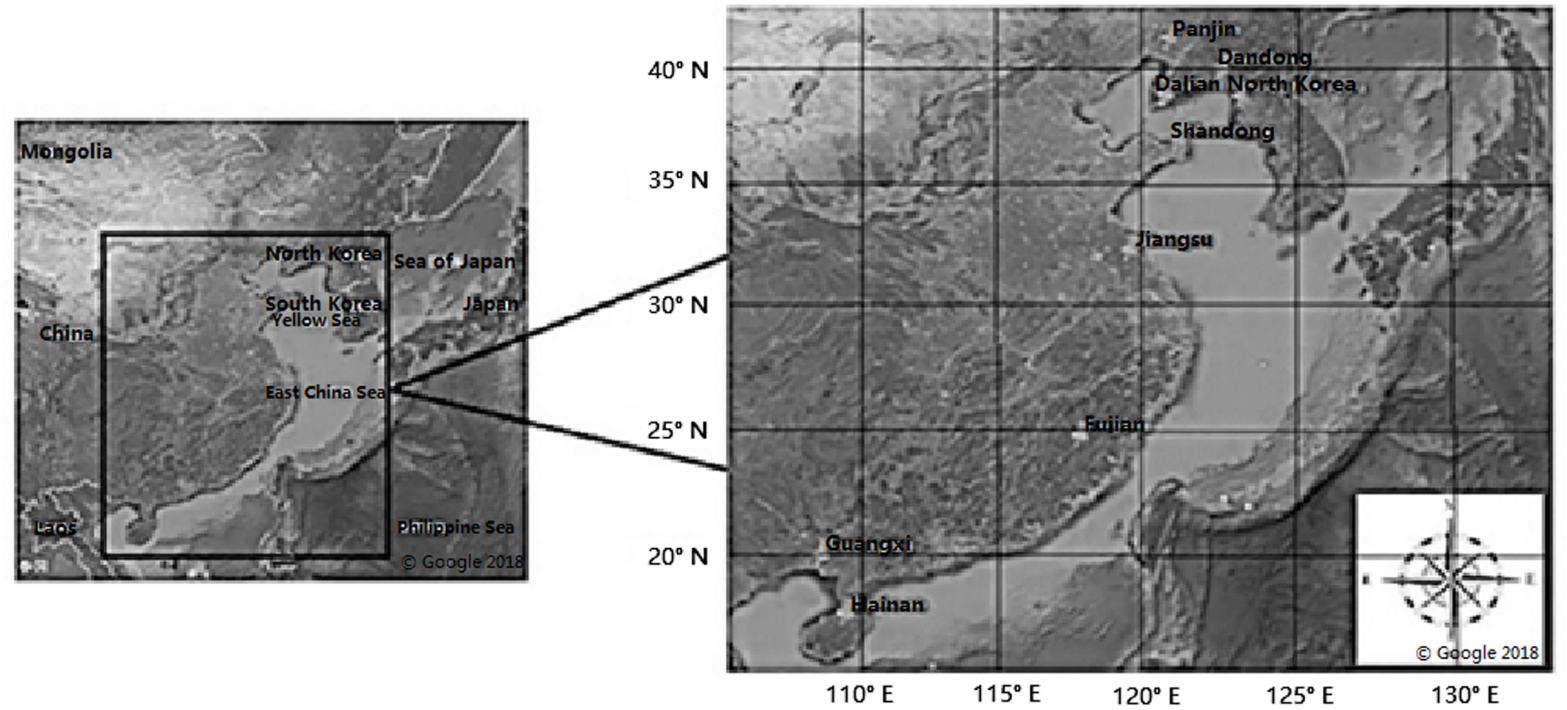
Maps showing locations of the nine populations of *Meretrix petechialis* sampled (FJ, GX, PJ, SD, DD, HN, DL, NK and JS). Maps showing locations of the nine populations of *Meretrix petechialis* sampled Fujian (FJ), Guangxi (GX), Panjin (PJ), Shandong (SD), Dandong (DD), Hainan (HN), Dalian (DL), North Korea (NK) and Jiangsu (JS). This map is attributed to Google map and the relevant data provider in Google Earth Pro on desktop Map data: Google, Maxar Technologies.

In this study, samples were collected from different tidal flats. A total of 270 samples were obtained from nine locations. We collected the clam *M. petechialis* at Fujian (FJ), Guangxi (GX), Panjin (PJ), Shandong (SD), Dandong (DD), Hainan (HN), Dalian (DL), North Korea (NK) and Jiangsu (JS) (Fig. 1). The adductor muscles were dissected from the samples and stored in 100% ethanol. DNA was extracted from the adductor muscles using a TIANamp Marine Animals DNA Extraction Kit (Tiangen, Beijing, China). After successful extraction, concentration was determined with 1% agarose gel electrophoresis and the quality of the extracted DNA was determined. The extracted DNA was stored in TE buffer and PCR was performed after the quality was confirmed. The diluted concentration was 100 ng/μL.

### Optimization of SRAP–PCR amplification system and detection of products

Primers for Me7 and Em15 were based on published SRAP sequence (Table 1), and 105 pairs of primers were obtained by crossing the upstream and downstream. The parameters of the PCR were as follows: 2.5 μL of 10× PCR buffer; two μL dNTP mixture; 0.25 μL Taq DNA polymerase produced by Takara, Japan; one μL upstream and downstream primers (10 μmol/L), and one μL template DNA, ddH_2_O to make up the volume of the PCR reaction to 25 μL. The reaction conditions were: 5 min at 94 °C, 5 cycles, 1 min at 94 °C, an annealing temperature of 35 °C for 1 min, an extension temperature of 72 °C, and an extension time of 1 min, 1 min at 94 °C, an annealing temperature of 50 °C for 1 min, extension at 72 °C for 1 min, a total of 35 cycles; extension at 72 °C for 10 min and stored at 4 °C. After the PCR was completed, it was detected by 6% denatured polyacrylamide gel electrophoresis. To ensure the accuracy of denatured polyacrylamide gel electrophoresis, a control was used for each test.

**Table 1:** Sequences of SRAP F-primer and R-primer.

Marker	R-primer	Marker	F-primer
*Em1*	GACTGCGTACGAATTAAT	*Me1*	TGAGTCCAAACCGGATA
*Em2*	GACTGCGTACGAATTTGC	*Me2*	TGAGTCCAAACCGGAGC
*Em3*	GACTGCGTACGAATTGAC	*Me3*	TGAGTCCAAACCGGAAT
*Em4*	GACTGCGTACGAATTTGA	*Me4*	TGAGTCCAAACCGGACC
*Em5*	GACTGCGTACGAATTAAC	*Me5*	TGAGTCCAAACCGGAAG
*Em6*	GACTGCGTACGAATTGCA	*Me6*	TGAGTCCAAACCGGTAA
*Em7*	GACTGCGTACGAATTCAA	*Me7*	TGAGTCCAAACCGGTAG
*Em8*	GACTGCGTACGAATTCTG		
*Em9*	GACTGCGTACGAATTCGA		
*Em10*	GACTGCGTACGAATTCAG		
*Em11*	GACTGCGTACGAATTCCA		
*Em12*	GACTGCGTACGAATTATG		
*Em13*	GACTGCGTACGAATTGTC		
*Em14*	GACTGCGTACGAATTACG		
*Em15*	GACTGCGTACGAATTAAG		

### SRAP band statistical analysis and calculation of genetic indicators

According to the SRAP amplification electrophoresis procedure, each of the bands is considered to be a locus, with reference to 10 bp DNA markers. The SRAP of each sample amplification band is counted, with a clearly visible band recorded as 1 and no band recorded as 0, and converted into 1 and 0 matrices. The polymorphism information content (PIC) value of the primer is calculated using PIC software. POPGENE32 (Yeh, Yang & Boylet, 1999) was used to observe the number of alleles (NA), the effective number of alleles (Ne), the Shannon’s information index (I), the Nei’s gene diversity (H), the number of polymorphic loci (N), and the percentage of polymorphic loci (P), the Nei’s unbiased genetic distance (D) and genetic similarity (S) were calculated and the neighbor-joining (NJ) tree of nine *M. petechialis* groups was constructed according to the genetic distance with MEGA 7.0 (Kumar, Stecher & Tamura, 2016). The total genetic variation among the samples was calculated using the phi-statistic through the analysis of molecular variance (AMOVA). This analysis was performed using the computer program GENALEX ver.6.5 (Peakall & Smouse, 2006). The total genetic variation is partitioned at three levels—within populations (Phi-PT), among populations within regions (Phi-PR) and among regional populations (Phi-RT) (Xu et al., 2008).

The patterns of the population structure were further investigated using the model-based Bayesian clustering procedure in STRUCTURE version 2.3.4 (Pritchard, Stephens & Donnelly, 2000), the data was converted into the appropriate format and imported into the software and relevant parameters were set. We assigned individuals to *K* populations based on their multilocus genotype. The estimated *K* value was set to 1–9 then STRUCTURE was used with 10,000 iterations and a burn-in period of 10,000. All runs were repeated 10 times at each *K*, the most appropriate value of *K* was identified with calculated Δ*K* by using Structure Harvester ver.0.6.92 (http://taylor0.biology.ucla.edu/structureHarvester/) (Earl & Vonholdt, 2012). Then, the optimal *K* value was independent run by the CLUMPP 1.1.2 (Jakobsson & Rosenberg, 2007), and the output was used as input into DISTRUCT 1.1 (Rosenberg, 2004) for cluster visualization. Furthermore, Principal Component Analysis (PCA) was employed to assess the degree of genetic relatedness among groups using Origin Pro.

## Results

### SRAP amplification band type

Using the genomic DNA of nine populations of *M. petechialis*, 105 pairs of SRAP primer combinations were screened, and 30 pairs of primer combinations with clear, stable and few bands were selected, of which eight pairs of primer combinations (*Me2/Em15*, *Me3/Em4*, *Me4/Em3*, *Me4/Em5*, *Me4/Em11*, *Me5/Em5*, *Me5/Em14*, *Me6/Em5*) can obtain stable, reproducible and polymorphic amplification maps. The polymorphic loci amplified by different primer combinations are different. The number of polymorphic loci detected by each pair of primers is 12–23, and the average number of loci per pair of primers is 16.5. The fragment size range of 116–250 bp has a high polymorphism, which indicates that SRAP can be applied to study genetic diversity in *M. petechialis*.

### Population genetic diversity

A total of 132 polymorphic loci were detected in eight pairs of primer combinations. The total stripe number was 308, of which the *Me5/Em14* polymorphic stripe number was the highest (23). The Me3/Em4 primer’s polymorphic frequency was the highest for 53.85% and the *Me6/Em5* was the lowest at 29.27% (mean = 42.86%). The PIC value of the *Me5/Em14* primer was the highest (0.90), the PIC value of the *Me4/Em5* primer was the lowest (0.78) and the average PIC value of eight pairs of primers was 0.86 (Table 2). The highest number of polymorphism bits and percentages of polymorphism sites in the JS population were 36% and 27.27% and the lowest in DL and NK populations were 32% and 24.24%, respectively. The Nei’s gene diversity of the nine *M. petechialis* populations ranged from 0.0647 (SD) to 0.0793 (NK) and the Shannon’s information index was between 0.1023 and 0.1202, with the SD population being the lowest and the JS population being the highest (Table 3).

**Table 2:** Polymorphism number and incidence for eight SRAP of *Meretrix petechialis*.

Primer combination	No. total bands	No. polymorphic band	Percentage of polymorphic band (%)	PIC
Me2/Em15	36.00	17.00	47.22	0.80
Me3/Em4	39.00	21.00	53.85	0.89
Me4/Em3	35.00	16.00	45.71	0.85
Me4/Em5	34.00	12.00	35.29	0.78
Me4/Em11	42.00	14.00	33.33	0.89
Me5/Em5	37.00	17.00	45.95	0.84
Me5/Em14	44.00	23.00	52.27	0.90
Me6/Em5	41.00	12.00	29.27	0.89
Total	308	132	–	–
Mean	–	–	42.86	0.86

**Table 3:** Polymorphism of SRAP amplified loci and genetic diversity parameter for nine population of *Meretrix petechialis*.

Pop	Sample size	*N*a	*N*e	*H*	*I*	*N*	*P* (%)
FJ	30	1.2652	1.1171	0.0732	0.1147	35	26.52
GX	30	1.2652	1.1193	0.0732	0.1144	35	26.52
PJ	30	1.2652	1.1242	0.0749	0.1156	35	26.52
SD	30	1.2652	1.1038	0.0647	0.1023	35	26.52
DD	30	1.2576	1.1242	0.0766	0.1183	34	25.76
HN	30	1.2576	1.1334	0.0785	0.1188	34	25.76
DL	30	1.2424	1.1275	0.0756	0.1149	32	24.24
NK	30	1.2424	1.1357	0.0793	0.1196	32	24.24
JS	30	1.2727	1.129	0.0779	0.1202	36	27.27

**Note:**

*N*a, Observed number of alleles; *N*e, Effective number of alleles; *H*, Nei’s gene diversity; *I*, Shannon’s Information index; *N*, The number of polymorphic loci; *P*, The percentage of polymorphic loci.

### Population genetic differentiation

**Figure 2:**
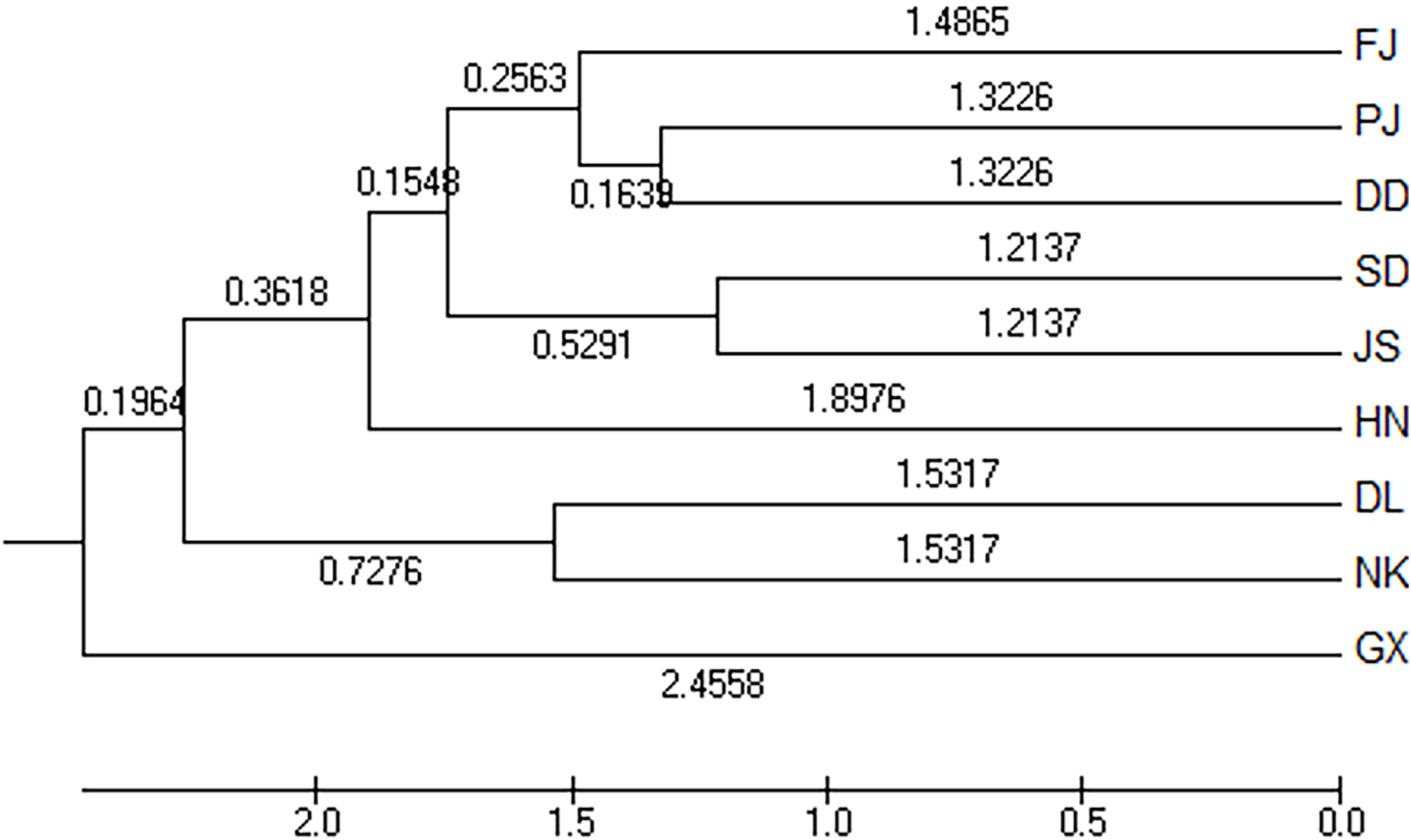
The unweighted pair group method with anarithmetic mean (neighbor-joining) dendrogram based on *D*_C_ distance among the nine populations of *Meretrix petechialis*.

The Nei’s unbiased measures genetic identity and genetic distance of nine geographical groups of *M. petechialis* were calculated according to the allele frequency of each locus and the results were shown in Table 4. The genetic distance between the GX and SD groups was the largest (0.0570) and the genetic similarity was the smallest (0.9446); The genetic distance between the SD and JS groups were the closest (0.0243) and the genetic similarity was the largest (0.9760). The cluster analysis of neighbor-joining trees showed that the GX forms a single population (Fig. 2), indicating that the genetic distance of the GX population was the furthest and there was a certain genetic differentiation.

**Table 4:** Nei’s unbiased measures of genetic identity and genetic distance.

pop	FJ	GX	PJ	SD	DD	HN	DL	NK	JS
FJ	****	0.9585	0.9725	0.9724	0.9689	0.9638	0.9675	0.9632	0.9629
GX	0.0424	****	0.9575	0.9446	0.959	0.9446	0.946	0.9508	0.9558
PJ	0.0279	0.0434	****	0.9606	0.9739	0.9606	0.9546	0.9559	0.9625
SD	0.0280	0.0570	0.0402	****	0.9631	0.9667	0.9554	0.9614	0.976
DD	0.0316	0.0418	0.0265	0.0376	****	0.9585	0.9463	0.9579	0.973
HN	0.0369	0.0570	0.0402	0.0339	0.0424	****	0.945	0.9548	0.9642
DL	0.0331	0.0556	0.0465	0.0457	0.0552	0.0566	****	0.9698	0.9449
NK	0.0375	0.0505	0.0451	0.0393	0.043	0.0463	0.0306	****	0.9582
JS	0.0379	0.0452	0.0382	0.0243	0.0273	0.0365	0.0514	0.0427	****

**Notes:**

Nei’s genetic identity (above diagonal) and genetic distance (below diagonal).

**** No value or null.

### Population clusters and structure analysis

**Figure 3:**
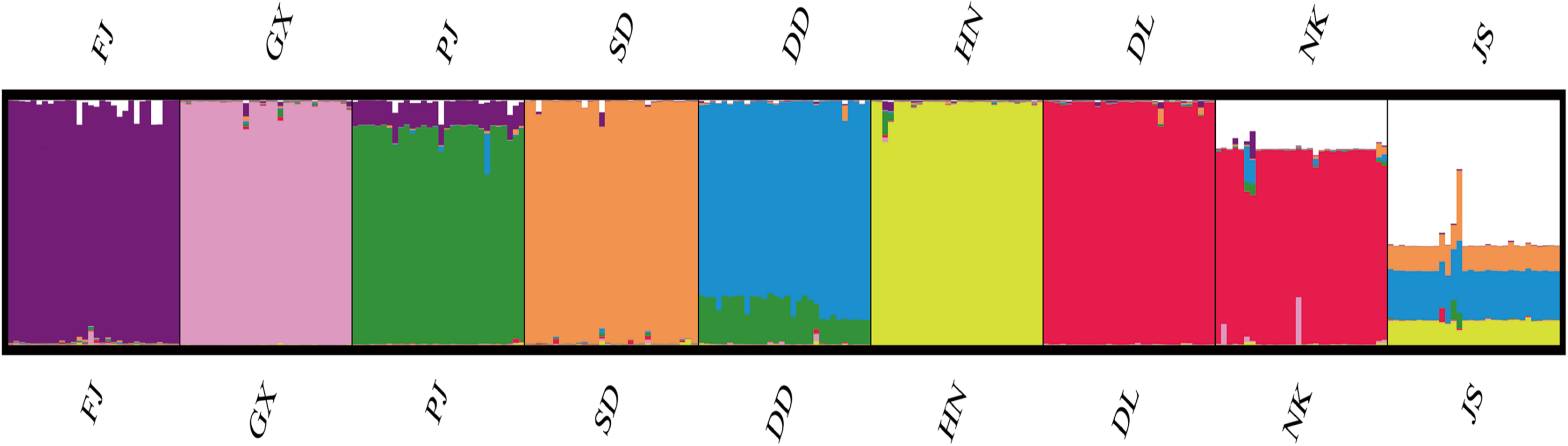
Genetic structure of 270 samples based on a mixed mode (cluster 1 purple is mainly FJ, cluster 2 pink is mainly GX, cluster 3 green is mainly PJ, cluster 4 orange is mainly SD, cluster 5 blue is mainly DD, cluster 6 yellow is mainly HN, cluster 7 red is mainly DL and NK, cluster 8 white is mainly JS).

**Figure 4:**
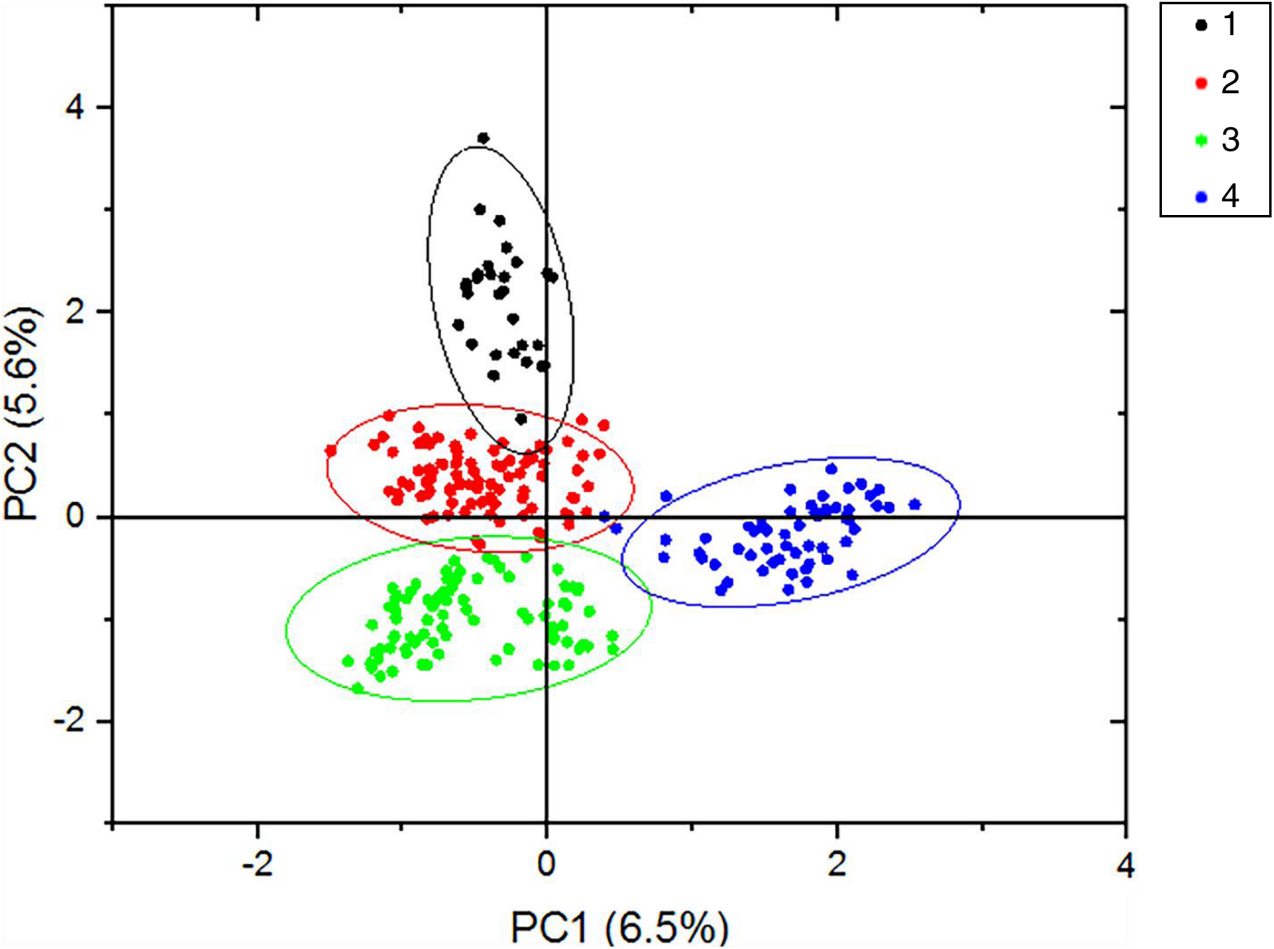
PCA results of the first two statistically significant components. The nine populations of *Meretrix petechialis* were split into 4 clusters (The first black circle includes GX; the second red circle includes FJ, PJ, DD; the third green includes SD, HN, JS; the last blue includes DL, NK).

According to the method of Earl & Vonholdt (2012), we used structure harvester website to analysis the *K*, the program provides a fast way to assess and visualize likelihood values across multiple values of *K* and thousands of iterations for easier detection of the number of genetic groups that best data. In the structure analysis, the highest Δ*K* = 8 (Fig. S1), which suggested that the nine populations of *M. petechial* were split into eight clusters (Fig. 3) and from the overall distribution of colors in it, we saw that most of the background colors of the DL and NK populations are the same, suggesting they were formed the same cluster, we suspected that it might be due to the close distance and similar genetic backgrounds. In contrast, the pink color which represented the GX group was hardly appeared in any other group, indicating that GX population has single genetic background, distant relationship and almost no gene flow with others populations. Additionally, the color composition of the remaining groups showed admixture with other populations color, it suggested that have a complex background and there is a certain degree of gene flow between the populations. The result was similar using the neighbor-joining dendrogram (Fig. 2). This also revealed that the northern and southern populations were not very integrated and their distribution pattern was could be based on geographical distance. To further elucidate the gene differentiation between populations, we performed (PCA) using Origin Pro. PCA was a supplement to cluster analysis, which analyzed the structural similarity of germplasm resources at marker locus. The PCA of 270 samples was calculated, in 132 principal components, the cumulative contribution rate of the first two principal components was 12.1%, contribution rates of the first and second principal components were 6.5% and 5.6%, respectively (Fig. 4). Nine populations were distributed into four different clusters, the PCA diagram revealed one cluster was GX population, two cluster included FJ, PJ, DD and three clusters included SD, HN, JS and four included DL, NK. In general, the classification results of each cluster have high correspondence with the NJ tree and structure. And there are many overlapping phenomena among samples in different geographical areas, but no obvious regional difference. However, because the cumulative contribution rate of the first two principal components is very low, which can reflect less genetic information and cannot accurately explain the genetic diversity information among populations. In this study, the AMOVA results were obtained from nine populations of *M. petechial* (Table 5). It revealed that populations within regions contributed to 10% of the total genetic variance (Phi-PR). The genetic variance within populations (Phi-PT) was 37% and 53% of the total genetic variance (PhiRT) was calculated between populations from different regions. This indicated that there was significant genetic differentiation within populations. The results obtained from the Nei’s genetic diversity analysis were consistent. The gene flow estimate obtained by PhiPT among populations (Nm (Haploid) = [(1/PhiPT)−1]/2) was 0.555, the Nm value was less than 1.0 and the number of gene exchanges between each generation was 0.555.

**Table 5:** Summary of the AMOVA results for 270 specimens of *Meretrix petechials*.

Source	df	SS	MS	Est. var.	Percentage (%)	Phi statistic	Value	*P* (rand ≥ data)
Among regions	7	1,182.239	168.891	1.113	10	PhiRT	0.099	0.001
Among pops	1	131.783	131.783	4.196	37	PhiPR	0.416	0.001
Within pops	261	1,538.133	5.893	5.893	53	PhiPT	0.474	0.001
Total	269	2,852.156		11.203	100			

**Note:**

df, degree of freedom; SS, sum of squared observations; MS, mean of squared observations; PhiRT, proportion of the total genetic variance that is, due to the variance between regions; PhiPR, proportion of the total genetic variance that is, due to the variance among populations within a region; PhiPT, proportion of the total genetic variance that is due to the variance among individuals within a variant.

## Discussion

At present, research on *M. petechialis* germplasm resources in China mainly adopts random amplified polymorphic DNA (RAPD) (Shen et al., 2003; Du et al., 2004), inter-simple sequence repeat (Chen et al., 2004), amplified fragment length polymorphism (AFLP) (Lin et al., 2008; Zhu et al., 2011). In addition, cytochrome oxidase I and internal transcribed spacer are widely used to reflect the genetic variation of genomic DNA from ribosomes to mitochondria (Li et al., 2006, 2016). To rapidly obtain *M. petechialis* genetic information, PCR-based SRAP marker technique was herein employed. Relative to other molecular markers, SRAP is simple, inexpensive, highly variable and less technically demanding to obtain or use and effective for producing genome-wide fragments with high reproducibility and versatility. Notably, SRAP markers target coding regions of the genome (Li & Quiros, 2001) and possess the capacity to elucidate markers with inherent biological significance (e.g., QTL identification), therefore have potential beyond more commonly applied multilocus markers. In present work, we collected samples from more regions, aiming to apply SRAP markers to grasp more diversity information of *M. petechialis* in recent years, so as to lay the foundation for genetic breeding.

Ecological and systematic studies often depend on the use of molecular tools to address questions regarding genetic relatedness among individuals, population structure and phylogenetic relationships. In the present study, SRAP markers were applied to assess the level and pattern of genetic diversity in nine populations of *M. petechialis*. The results show that 132 polymorphic bands were detected by eight pairs of SRAP primers and the number of polymorphic alleles with an average amplification of each pair of primers was 16.5. Compared with the results of the study that analyzed the germplasm resources in three geographical groups of *M. petechialis* from Guangxi Beihai (GX), Jiangsu Nantong (JS) and Liaoning Dalian (LN) by SRAP markers (Zhang et al., 2010), although the average polymorphism of each primer detected was low, the average number of bands detected by each primer was relatively high, indicating that even if there were various differences in the test, the diversity of the samples detected by the SRAP marker was stable and reliable.

Genetic diversity is an important indicator of population genetic variation, mainly reflecting changes in genetic differences between different genetic loci. Among them, Nei’s gene diversity and Shannon’s information index are common indicators reflecting genetic diversity among populations. According to the polymorphism of SRAP amplified loci and genetic diversity parameter for nine population of *M. petechialis*, the JS population has a abundant genetic diversity, which was similar to previous studies reported by Shen et al. (2003), Chen et al. (2004) and Li et al. (2016).

In this study, we applied Structure, PCA and NJ tree to further analyze and illustrate the genetic diversity of nine geographical *M. petechialis* populations. Combining the results of three different analysis, GX forms a single population indicated that the genetic distance of the GX population with other populations was the largest and the germplasm resources of GX are relatively pure and there was no cross-contamination with other groups but a certain genetic differentiation, which is consistent with previous study using AFLP markers (Lin et al., 2008) and SRAP markers (Zhang et al., 2010). The local germplasm resources in FJ, GX, SD, HN, DL populations were better protected with the population invasion from other areas is relatively mild (Fig. 3). In addition, due to different place transplantation, proliferation and release, the genetic background of PJ, DD, NK, JS populations were polluted by other populations to varying degrees. The genetic similarities of the nine geographical *M. petechialis* populations were all less than 1, but they could be clustered together, indicating that the populations have similar genetic backgrounds, but there are certain genetic differences.

The results of this study elucidated that the genetic diversity and population polymorphism is relatively high in GX population, therefore, the potential for the genetic improvement of the breed is in GX population. Nei’s gene diversity analysis indicated that the genetic variance was mainly found within individual geographical populations and the AMOVA revealed low but significant genetic differentiation among local and regional populations. The limited gene flow (Nm = 0.555) was inferred as a major reason for the extent of genetic differentiation in *M. petechialis*. Wright (1931) suggested that when the Nm value was less than 1.0, the limited gene flow is mainly the reason for the genetic differentiation of the populations and the gene differentiation caused by genetic drift cannot be effectively suppressed (Qu et al., 2004).

In recent years, due to the continuous reduction of *M. petechialis* germplasm resources, its genetic background has become unstable. The choice of breeding is the main method to improve the beneficial traits of *M. petechialis*. At the same time, genetic structure analysis of *M. petechialis* can be carried out by developing and applying molecular techniques to provide a scientific basis for the genetic breeding of *M. petechialis*. The results obtained here indicated that *M. petechialis* have high degree of genetic diversity and the potential of further breeding with excellent germplasm resources. The GX population might be more suitable as the base population for selective breeding because its genetic improvement potential is the highest.

## Conclusions

In the present study, different geographical populations of *M. petechialis* were analyzed by SRAP molecular marker to estimate the genetic differentiation and population structure. Nei’s gene diversity analysis indicated that the genetic variance was mainly found within individual geographical populations and the AMOVA revealed low but significant genetic differentiation among local and regional populations. The limited gene flow was inferred as a major reason for the extent of genetic differentiation in *M. petechialis*. The GX population might be more suitable as the base population for selective breeding because its genetic improvement potential is the highest.

## Supplemental Information

10.7717/peerj.8723/supp-1 Supplemental Information 1 Curve diagram of Δ*K* value.

10.7717/peerj.8723/supp-2 Supplemental Information 2 The amplification results of Primer Me5, Em5, Me5, Em14 and Me6, Em5 by PAGE electrophoresis.

10.7717/peerj.8723/supp-3 Supplemental Information 3 The original band pattern of PCR products of Me2, Em15, Me3, Em4, Me4, Em3, Me4, Em5 and Me4, Em11.

10.7717/peerj.8723/supp-4 Supplemental Information 4 The gynotypes of different SRAP markers at nine populations of *Meretrix petechialis*.

## Additional Information and Declarations

### Data Availability

The following information was supplied regarding data availability: The raw data is available in the Supplemental Files.

## References

[ref-1] Chen DP, Shen HS, Ding YP, Yang JX, Shen SD, Xu P (2004). Using inter-simple sequence repeats (ISSR) technique in two populations of *Meretrix meretrix*. Journal of Nanjing Normal University.

[ref-2] Ding WD, Cao LP, Cao ZM (2008). The SRAP and SCAR molecular markers for detecting germ degeneration in *Ctenopharyngodon idellus*. Acta Zoologica Sinica.

[ref-3] Du XD, Deng YW, Ye FL, Wang H (2004). Genetic diversity of seven wild populations of meretrix meretrix. Journal of Fishery Sciences of China.

[ref-4] Earl DA, Vonholdt BM (2012). Structure harvester: a website and program for visualizing structure output and implementing the Evanno method. Conservation Genetics Resources.

[ref-6] Jakobsson M, Rosenberg NA (2007). CLUMPP: a cluster matching and permutation program for dealing with label switching and multimodality in analysis of population structure. Bioinformatics.

[ref-7] Jin D, Qian YJ, Yan XW, Li X, Zhao LQ (2013). Establishment of SRAP reaction conditions and the genetic analysis of three strains with different shell colors in Manila clam *Ruditapes philippinarum*. Journal of Dalian Ocean University.

[ref-8] Kumar S, Stecher G, Tamura K (2016). MEGA7: molecular evolutionary genetics analysis version 7.0 for bigger datasets. Molecular Biology Evolution.

[ref-9] Li G, Quiros CF (2001). Sequence-related amplified polymorphism (SRAP), a new marker system based on a simple PCR reaction: its application to mapping and gene tagging in *Brassica*. Theoretical and Applied Genetics.

[ref-10] Li TW, Zhang AG, Su XR, Li CH, Liu BZ, Lin ZH, Chai XL (2006). The analysis of ITS2 in *Meretrix meretrix* with different stripes. Oceanologia et Limnologia Sinica.

[ref-11] Li HJ, Zhang JJ, Yuan XT, Zhang AG, Liu GZ, Shao KS, Wang LJ (2016). Genetic diversity and differentiation of seven geographical populations of hard clam (*Meretrix meretrix*) assessed by COI and microsatellite markers. Acta Ecologica Sinica.

[ref-12] Lin ZH, Dong YH, Li N, Lu RM, Xiao GQ, Chai XL, Liu BZ, Sun CS, Bao ZM, Hu JJ (2008). The genetic structure and diversity analysis of different geographical populations of *Meretrix meretrix* using morphol ogical parameters and AFLP markers. Oceanologia Et Limnologia Sinica.

[ref-13] Peakall R, Smouse PE (2006). Genalex 6: genetic analysis in excel population genetic software for teaching and research. Molecular Ecology Resoures.

[ref-14] Pritchard JK, Stephens M, Donnelly P (2000). Inference of population structure using multilocus genotype data. Genetics.

[ref-15] Qiao H, Wu Y, Fu H, Gong Y, Jiang S, Xiong Y (2012). Construction of a genetic linkage map for oriental river prawn (*Macrobrachium nipponense*) using SSR and SRAP markers. Journal of Fishery Sciences of China.

[ref-16] Qu RZ, Hou L, Lv HL, Li HY (2004). The gene flow of population genetic structure. Hereditas.

[ref-17] Rosenberg NA (2004). Distruct: a program for the graphical display of population structure. Molecular Ecology Resources.

[ref-18] Shen HS, Zhu JP, Ding YP, Chen GY, Lu QQ, Zhu MX, Xu P (2003). RAPD analysis of three wild stocks of *Meretrix meretrix* in China’s coastal seas. Acta Oceanologica Sinica.

[ref-19] Tang BP, Zhou K, Song D, Yang G, Dai A (2003). Molecular systematics of the Asian mitten crabs, genus Eriocheir (Crustacea: *Brachyura*). Molecular Phylogenetics and Evolution.

[ref-20] Wen HB, Cao ZM, Jin W, Gu RB, Hua D, Huang XF, Xu P (2014). Analysis of differential gene expression by SRAP-cDNA in mantle tissue of *Hyriopsis cumingii* with different external shell color. Acta Hydrobiologica Sinica.

[ref-21] Wright S (1931). Evolution in mendelian populations. Genetics.

[ref-22] Xu JP, Sha T, Li YC, Zhao ZW, Yang ZL (2008). Recombination and genetic differentiation among natural populations of the ectomycorrhizal mushroom Tricholoma matsutake from southwestern China. Molecular Ecology.

[ref-23] Yeh FC, Yang RC, Boylet (1999). POPGENE version 1.32 microsoft window-based freeware for population genetic analysis [CP]. https://sites.ualberta.ca/~fyeh/fyeh/.

[ref-24] Zhang ZW, Chen AH, Yao GX, Wu JP, Wu YP, Xu GP, Cheng HL (2010). SRAP analysis on germplasm of wild *Meretrix Meretrix* off Chinese coasts. Oceanologia Et Limnologia Sinica.

[ref-25] Zheng JH, Nie HT, Yan XW (2019). Analysis of differential gene expression by SRAP-cDNA in mantle tissue of *Meretrix petechialis* with different external shell color. Epub ahead of print 22 July 2019. Animal Biotechnology.

[ref-26] Zhu DL, Lin ZH, Dong YH, Yao HH (2011). Genetic variation analysis of four strains of *Meretrix Meretrix* that have different shell colors and decorative patterns. Oceanologia Et Limnologia Sinica.

