# Supplementary figures and images for: Genetic diversity and population structure of Meretrix petechialis in China revealed by sequence-related amplified polymorphism markers
Source: PeerJ. 2020 Mar 25;8:e8723. doi: 10.7717/peerj.8723 (PMC7102500; doi:10.7717/peerj.8723)

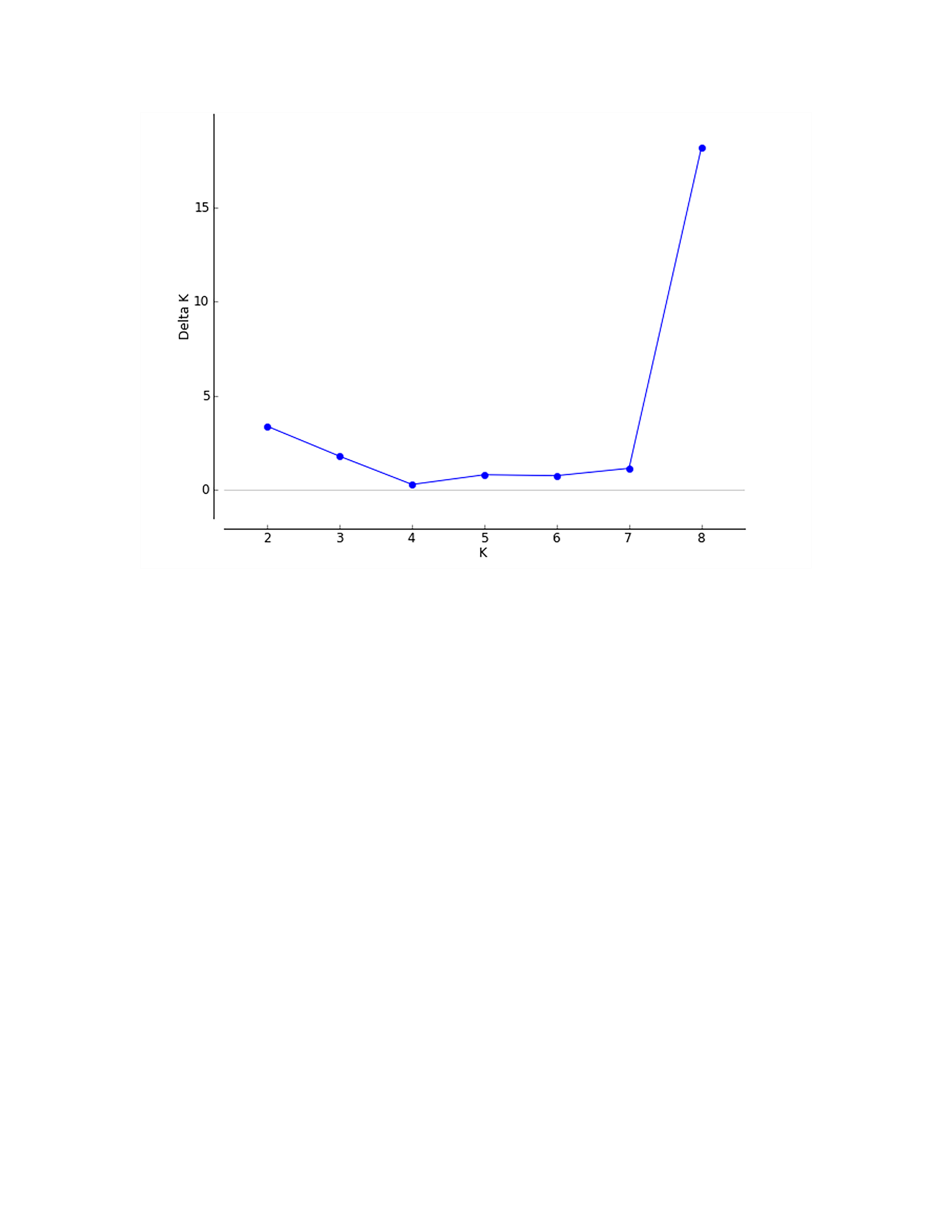

Supplement: Supplemental Information 1 [file peerj-08-8723-s001.png]
